# Adequate bowel preparation of patients undergoing screening colonoscopy: Does educational status matter? A retrospective data analysis

**DOI:** 10.1007/s00508-024-02468-5

**Published:** 2024-12-10

**Authors:** Anna K. Hinterberger, Susanne Mayer, Lena Jiricka, Elisabeth A. Waldmann, Jasmin Zessner-Spitzenberg, Barbara Majcher, Lisa M. Rockenbauer, Michael H. Trauner, Monika Ferlitsch

**Affiliations:** 1 Quality Assurance Working Group, Austrian Society for Gastroenterology and Hepatology, Vienna, Austria; 2 Department of Health Economics, Center for Public Health, Medical University of Vienna, Vienna, Austria; 3 Division of Gastroenterology and Hepatology, Department of Internal Medicine III, Medical University of Vienna, Waehringer Guertel 18–20, 7i, 1090 Vienna, Austria

**Keywords:** Patient education, Socioeconomic status, Bowel prerperation, Health literacy, Screening colonoscopy

## Abstract

**Background:**

In order to achieve a high-quality screening colonoscopy, a high-quality bowel preparation is essential. To perform an adequate bowel cleansing patients need to understand and act on medical information, also known as health literacy. This study aimed to analyze the relationship between the patients’ educational status as a proxy for health literacy and adequate bowel preparation.

**Methods:**

A retrospective analysis of data collected within the Austrian national colorectal cancer screening program from 2012 to 2022 was conducted. The database contains information on the quality of bowel preparation as well as the academic degree of the patients. Variables were used to perform a logistic regression analysis, with bowel preparation quality (based on the Aronchick scale) as the dependent variable and completed tertiary education as independent variable, and sex and age as control variables.

**Results:**

A total of 329,778 patients aged 30–99 years were included in the analysis. Within the group of academics, 88.46% (*n* = 21,883) were adequately prepared whereas in the group of non-academics, 84.79% (*n* = 258,641) were found to have a high bowel preparation quality. The odds ratio (OR) for academics to have an adequate bowel preparation quality was 1.37 (95% confidence interval, CI 1.32–1.43; *p* < 0.001).

**Conclusion:**

Patients with a tertiary education showed better bowel preparation quality than non-academics in Austria. Therefore, it is important to improve strategies on how to inform also less educated persons to facilitate a better screening colonoscopy quality and to optimize the use of resources from a clinical as well as a public health perspective.

**Supplementary Information:**

The online version of this article (10.1007/s00508-024-02468-5) contains supplementary material, which is available to authorized users.

## Introduction

Colorectal cancer is the third most common cancer worldwide with 1,931,590 new cases registered in 2020 [1]; however, colorectal cancer can be detected at an early stage and thus prevented [2]. The gold standard examination for detecting precancerous lesions is screening colonoscopy. During the screening colonoscopy, the mucosa of the colon is visualized from the rectum to the cecum and even further to the terminal ileum using an endoscope [3, 4]. As this examination is performed on a healthy population and electively, a high quality is essential to minimize the risk of, for example, bowel perforation as well as to avoid repetition of the examination to keep resource use to a minimum [5].

In Austria, screening colonoscopy is recommended and financed by the Austrian statutory health insurance from the age of 50 years [3], with costs amounting to approximately € 350 per examination, according to a regional study [6]. For quality assurance of the screening colonoscopy the guidelines of the Austrian Society of Gastroenterology and Hepatology as well as the guidelines of the European Society of Gastrointestinal Endoscopy (ESGE) are applicable in Austria [7, 8]. These guidelines list quality criteria which should be fulfilled before, during and after the examination (follow-up criteria). One of the criteria is an adequate bowel preparation. Adequate bowel preparation is important for the examiner to be able to visualize the whole mucosa, to advance the endoscope to the cecum and detect and remove precancerous lesions [7–9].

To achieve a clean bowel, the patient must take a laxative powder with about 2l of water. Patients usually take the laxative at home, except when the patient, for instance, is an inpatient in the hospital. It is also recommended to take the laxative in a split dose, i.e., one dose the day before the examination and the second dose in the morning before the examination. In addition, certain foods should not be consumed on the day of the examination and the day before the examination [8, 9]. Information on the necessary preparation is usually provided by a nurse or the examiner, i.e., the endoscopist, when explaining the examination procedure, the time interval between explanation and examination should be kept as short as possible and should be repeated after 6 months if colonoscopy does not take place within the time frame of 6 months [10]. Flyers and information brochures may also be handed out to patients [9]. For each endoscopist there should be an adequate bowel preparation in at least 90% of the examined patients according to the guidelines [5, 7, 8]. To evaluate the adequate bowel preparation rate, several scales are available to assess the cleanness of the bowel [14], such as the Boston bowel preparation scale and the Aronchick scale. It is up to the examiner which one to use [9, 10].

For patients to achieve an adequate bowel preparation, they should be informed as precisely as possible and adhere to the physician’s instructions. At the same time, compliance by the patients requires proper understanding and ability to act on the information provided or put differently, a certain level of health literacy [11]. A Dutch study showed that health literacy is lower in a population with low socioeconomic status and especially with low educational status [12]. A US study investigating risk factors for noncompliance of patients performing split-dose bowel preparation determined that 15.7% of compliant patients had a high school diploma or less versus 18.8% of noncompliant patients; however, these differences were not statistically significant [13]. Another US study concluded that patients have worse colonoscopy preparation if they are older, have diabetes, a prior abdominal history or government sponsored insurance [14].

Against this background with few recent studies in this area and no evidence available specifically for Austria, this study aims to determine whether there is an association between adequate bowel preparation and educational status as a potential marker for health literacy. Such findings may highlight the potential need for optimizing information material or methods to educate the relevant population. Accordingly, the quality of future screening colonoscopy could be improved and the use of economic and medical resources optimized as any screening colonoscopy with poor preparation quality is not sufficient and has to be repeated.

## Methods

Data for this study were obtained within the “Certificate of quality for screening colonoscopy”, which is a national screening program in Austria with the aim to measure and optimize quality of screening colonoscopies [4]. The program was founded in 2007 in cooperation with the Austrian Society of Gastroenterology and Hepatology, the Main Association of Social Security Institutions and the Austrian Cancer Aid. Participants of the program are gastroenterologists throughout Austria, currently (as of 2023) about 50% of Austrian gastroenterologists participate in this program. The participating physicians perform endoscopies in hospitals, outpatient clinics and in private practices. To participate in the program, physicians must meet certain criteria including i) being a specialist in internal medicine or a specialist in surgery with proven performance of at least 200 supervised colonoscopies (up to the cecum) and at least 50 supervised polypectomies; ii) at least 100 independently performed colonoscopies (up to the cecum) and at least 10 independently performed polypectomies per year.

As part of the Austrian program, physicians who perform screening colonoscopies fill out an online form that gathers relevant information about the examination and other patient-related and physician-related characteristics. For this study, the two most relevant variables included in the database are the bowel preparation quality (as drop-down field) assessed based on the Aronchick scale [11]. The patient’s preparation quality can be either “excellent”, “good”, “fair”, “poor” or “insufficient”. In our analysis, bowel preparation classified as “excellent”, “good” and “fair” was summarized as “adequate”, while “poor” and “insufficient” were coded as “inadequate” bowel preparation in line with the methodology to determine the adequate bowel preparation rate per examiner [5].

The other key variable for this study is the patient’s academic degree (as an open question) as an indicator for the patient’s level of education. In the online form, there are two fields for the academic title: “title in front” and “title in back”, as in Austria, some titles are put before the name (e.g. Ing.) and some after (e.g. Msc.). Both types of titles were evaluated based on the International Standard Classification of Education (ISCED) scheme. For our analysis, ISCED 6–8 was defined as academic degree, with ISCED 6 including bachelor’s degrees, ISCED 7 master’s degrees and ISCED 8 including doctor’s degrees or PhDs [15]. All patients with no titles stated or titles at ISCED level up to 5 were defined as nonacademic. The same applies to titles which were unclear due to assumed typing mistakes such as “q” or “sen” (0.02%, *n* = 75).

The field on bowel preparation quality has been part of the online form since 2012, so data from 2012 to 2022 were analyzed in this study. Within the database only patients who meet the screening criteria are included, i.e., patients who do not have any chronic intestinal diseases, are older than 30 years (when having screening characteristics), but not older than 100 years, and are not multimorbid.

All patients who were included in this study signed a consent form to agree with the retrospective analysis of their pseudonymized data and publication. The study was approved by the ethics committee of the Medical University of Vienna (1932/2022).

### Study population and statistical analysis

Information on exclusion of patients for this study is presented in Figure S1 (see supplementary material). Firstly, data on patients with no information on bowel preparation, including entries before 2012 or incomplete entries due to errors in data transmission (21.76%, *n* = 96,778) were excluded. Secondly, patients younger than 30 were excluded as these patients do not meet the screening criteria (0.19%, *n* = 834). Thirdly, only the first screening colonoscopy was analyzed, repeated colonoscopies were excluded (3.88%, *n* = 17,253) as patients might be more knowledgeable after experiencing the procedure once.

Characteristics of the study population by sex and academic background for the included patients and for the Austrian population-based data of Statistics Austria to enable a comparison are presented as percentages with the latter focusing on the target group of individuals aged 45 years and older [16, 17].

To determine the association between bowel preparation quality and academic status, a logistic regression analysis was performed using Stata Version 17 (DPC Software GmbH, Solingen, Germany), with bowel preparation quality as dependent variable and educational level (academic degree) as the independent variable. Age and gender were included as control variables. Odds ratios (OR) and 95% confidence intervals (CI) were calculated. To obtain robust confidence intervals in light of repeated observations of physicians, the sandwich estimator was employed.

## Results

### Descriptive characteristics of the study participants in the screening program compared to the Austrian screening target population

With 7.5% (*n* = 24,738), the proportion of patients with tertiary education who underwent screening colonoscopy is lower than the respective proportion in the general target population (12.23%). Table 1 shows the patient characteristics of the included study participants by educational status and Table 2 presents the characteristics of the Austrian target population for screening colonoscopy based on data from Statistics Austria for comparison.

**Table 1:** Description of study population who underwent a screening colonoscopy between 2012 and 2022 (*n* = 329,778)

Characteristics	Total (*n* = 329,778)	Academics (*n* = 24,738; 7.50%)	Nonacademics (*n* = 305,040; 92.50%)
Male, *n* (%)	160,460 (48.66%)	16,908 (68.35%)	143,552 (47.06%)
Female, *n* (%)	169,318 (51.34%)	7829 (31.65%)	161,489 (52.94%)
Mean age, years (standard deviation)	66.76 (10.11)	64.34 (9.60)	66.97 (10.14)

*Source*: Own calculation based on the “Certificate of quality for screening colonoscopy” database [4]

**Table 2:** Description of target population* for screening colonoscopy in the general population (*n* = 4,220,176; aged 45–99 years)

Characteristics	Total (*n* = 4,220,176)^1^	Academics (*n* = 516,134^1^; 12.23%)	Nonacademics (*n* = 3,704,042^2^; 87.77%)
Male, *n* (%)	1,994,779^1^ (47.27%)	262,926^1^ (50.93%)	1,731,853^2^ (46.75%)
Female, *n* (%)	2,225,397^1^ (52.73%)	253,208^1^ (49.07%)	1,972,189^2^ (53.25%)

*defined as people 45 years of age and not older than 99 years

^1^: Statistic Austria, “Population/level of education 2019” [16, 17]

^2^: Own calculation based on data from Statistic Austria “Population 2019” [16, 17]

As shown in Table 3, within the group of academics 88.46% (*n* = 21,883) were adequately prepared whereas 11.54% (*n* = 2855) were not adequately prepared for the colonoscopy. Within the group of nonacademics, 84.79% (*n* = 258,641) had a high bowel preparation quality compared to 15.21% (*n* = 46,399) with poor bowel preparation quality.

**Table 3:** Quality of bowel preparation in academics and nonacademics (*n* = 329,778)

	Academics (*n* = 24,738)	Nonacademics (*n* = 305,040)
Adequate bowel preparation (*n* = 280,524)	*n* = 21,883 (88.46%)	*n* = 258,641 (84.79%)
Inadequate bowel preparation (*n* = 49,254)	*n* = 2855 (11.54%)	*n* = 46,399 (15.21%)

*Source*: own calculation based on database of “Certificate of quality for screening colonoscopy” [4]

### Association between educational status and quality of bowel preparation

The OR for academics having an adequate preparation quality of the bowel compared to nonacademics is 1.372 (95% CI = 1.318–1.429; *p* = 0.0001). With respect to the control variables, female sex is associated with a OR for adequate bowel preparation, and older age with a lower preparation quality (Table 4).

**Table 4:** Association between bowel preparation quality and tertiary education (*n* = 329,778)

Category	OR for having an adequate bowel preparation	Confidence interval (95%, upper-lower)	*p*-value
ISCED level ≥ 6	1.372	1.316–1.429	0.0001
Female	1.203	1.181–1.227	0.0001
Age	0.987	0.986–0.988	0.0001
Constant	11.005	10.303–11.756	0.0001

*OR* odds ratio, *ISCED* International Standard Classification of Education

*Source*: own calculation based on database of “Certificate of quality for screening colonoscopy” [4]

## Discussion

This study is so far the first investigation including 329,778 patients undergoing screening colonoscopy analyzing the association between bowel preparation quality and educational status in Austria in several centers (hospitals, private practice).

In this Austrian study, patients with an academic degree were found to have a higher chance of having an adequate bowel preparation compared to patients without an academic degree. This finding could be related to the fact that patients with a higher level of education tend to have a higher health literacy [18]. The assumption of higher education level being associated with higher health literacy is in line with results of an earlier Austrian study concluding that education is positively correlated with the health literacy index [19]. Another study found health literacy in Austria to be generally lower compared to other European countries [20]. The health literacy was inadequate in 18.2% of the Austrians and problematic in 38.2%, compared to other countries, Austria thus has the third rank of the worst health literacy after Bulgaria and Spain [20]. Therefore, Austrians generally may need more or better explanation of health-related information. Furthermore, in a population with people aged 66–75 years health literacy was also found to be limited [20]. Thus, patients who undergo screening colonoscopy might be more likely to have a worse health literacy due to their higher age.

Some studies have already investigated the association between bowel preparation and sociodemographic data, including low educational status but results varied widely and numbers of included patients were low, therefore, the influence of sociodemographic data on bowel preparation is not clarified [21]. Nevertheless, studies show that certain social factors have an influence on health literacy. A US study indicated that especially older adults without a high school diploma, therefore with a low educational status, have a lower health literacy and are 2.4 times more likely to express a fair or poor health status compared to the group with a high school diploma (48% vs. 20%) [22]. Another US study showed that health literacy positively correlated with adherence in medication or nonmedication. Also, health literacy interventions had a significant effect on the health literacy in groups with lower income and of racial-ethnic minority patients [23].

The fact that people with no academic degree have a higher chance of having an inadequate bowel preparation shows that there is a need for improved information transfer in these groups to achieve a high-quality screening colonoscopy and reduce the risk of developing colorectal cancer. Morbidity and mortality in a group with a low socioeconomic status have previously been found to be higher than in the group of people with a higher socioeconomic status and therefore also morbidity and mortality depend on the level of education [24]. Thus, there is a health gap between people with a high educational level and a low educational level. To close this gap, it might need policy decisions on optimization strategies [24]; however, optimization should not only take place in a public health perspective but also at a clinical level. To prevent poor preparation quality in people with a lower educational status, information material on screening colonoscopy and educational strategies should be critically reviewed and optimized if necessary. Educational material in Austria is often provided in written form and could be made easier to understand with more pictures. Earlier studies already show that there are differences in the quality of information material [25]. Furthermore, structured patient education strategies help to improve colon preparation quality, as for example showing a short video on bowel preparation [26]; however, the number of studies on quality differences between verbal, written and pictorial information for patients is very limited. Pelikan et al. published a concept where nine suggestions for improving health literacy in Austria are mentioned, for example people with low health literacy should be included in the development and evaluation process of e.g., information material [27].

The findings of this study need to be interpreted in the light of some limitations. Firstly, there were more nonacademics in our database than academics, even though there are more nonacademics than academics in the general Austrian population. This could be due to the fact that physicians complete the patient data in the online form and the field “title” is not mandatory. This implies that some academics may have been mistakenly classified as nonacademics, potentially introducing a downward bias on the reported findings. From the side of the patients, it may also be the case that they do not mention their title in the first place. At the same time, an earlier study showed that in international comparison, Austrians are more likely to use and state titles [28]. Secondly, particularly in hospitals, the physicians who provide information on adequate bowel preparation may alternate, so that the endoscopists who perform the procedures do not necessarily have to be the physician who provides the information. Thus, it would be possible that there is a lack of motivation to emphasize the importance of adequate bowel preparation, as the explaining gastroenterologist does not conduct the colonoscopy and might have limited time resources. To counteract this possible variation of education quality, nurse-assisted patient education programs, as already implemented at the Division of Gastroenterology and Hepatology at Medical University of Vienna, might help. Within these programs special nurses only perform the patient education, therefore the provision of information could be more efficient and might have a higher quality. As in this study no data from the Medical University of Vienna are included, further investigations should be conducted as they might show a difference in the quality of bowel preparation when comparing centers with a nurse-assisted patient education program versus centers where patients are educated by physicians.

## Conclusion

Nonacademics in Austria who undergo screening colonoscopy have a higher risk for inadequate bowel preparation. Therefore, in the field of screening colonoscopy, future studies should investigate how to best convey the correct performance of bowel preparation to different population groups.

### Bullet key points

- Patients undergoing screening colonoscopy should have an adequate bowel preparation to detect premalignant lesions and reduce colorectal cancer incidences.
- Nonacademics in Austria who undergo screening colonoscopy have a higher risk for having inadequate bowel preparation.
- Information material for patients should be designed to be easily understood by every patient, regardless of the educational status.
- A consistent and adequate educational method for bowel preparation should be chosen in every clinic to achieve a unified patient education (for example, through nurse-assisted education programs).

## Supplementary Information

Figure S1: Patient exclusion criteria (see supplementary material)

## Data Availability

The data that support the findings of this study are available in anonymized form on request from the corresponding author Prof. Dr. Monika Ferlitsch.

## Abbreviations

CI: Confidence interval
ESGE: European Society of Gastrointestinal Endoscopy
ISCED: International Standard Classification of Education
OR: Odds ratio
