# Supplementary figures and images for: Adequate bowel preparation of patients undergoing screening colonoscopy: Does educational status matter? A retrospective data analysis
Source: Wien Klin Wochenschr. 2024 Dec 10;137(17-18):557–62. doi: 10.1007/s00508-024-02468-5 (PMC12446085; doi:10.1007/s00508-024-02468-5)

**Figure S1:** Patient exclusion criteria

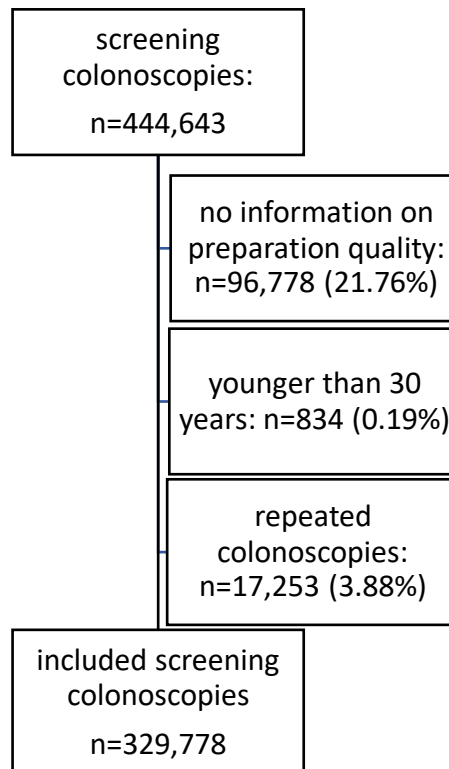

Supplement: Supplementary file 1 — Figure S1: Patient exclusion criteria (see supplementary material) [file 508_2024_2468_MOESM1_ESM.pdf]
